# Intratumoral in vivo staging of breast cancer by multi-tracer PET and advanced analysis

**DOI:** 10.1038/s41523-022-00398-x

**Published:** 2022-03-24

**Authors:** Jennifer Griessinger, Julian Schwab, Qian Chen, Anna Kühn, Jonathan Cotton, Gregory Bowden, Heike Preibsch, Gerald Reischl, Leticia Quintanilla-Martinez, Hidetoshi Mori, An Nguyen Dang, Ursula Kohlhofer, Olulanu H. Aina, Alexander D. Borowsky, Bernd J. Pichler, Robert D. Cardiff, Andreas M. Schmid

**Affiliations:** 1 Werner Siemens Imaging Center, Department of Preclinical Imaging and Radiopharmacy, Eberhard Karls University Tuebingen, Tuebingen, Germany; 2 Institute of Medical Systems Biology, Ulm University, Ulm, Germany; 3 Center for Immunology and Infectious Diseases, University of California, Davis, CA USA; 4 Department of Radiology, University Hospital Tuebingen, Tuebingen, Germany; 5 Cluster of Excellence iFIT(EXC 2180) “Image-Guided and Functionally Instructed Tumor Therapies”, University of Tuebingen, Tuebingen, Germany; 6 Department of Pathology, Eberhard Karls University Tuebingen, Tuebingen, Germany; 7 Janssen Pharmaceutical, Spring House, PA USA; 8 German Cancer Consortium (DKTK), Partner Site Tuebingen; German Cancer Research Center (DKFZ), Heidelberg, Germany

**Keywords:** Breast cancer, Preclinical research, Cancer imaging, Diagnostic markers

## Abstract

The staging and local management of breast cancer involves the evaluation of the extent and completeness of excision of both the invasive carcinoma component and also the intraductal component or ductal carcinoma in situ. When both invasive ductal carcinoma and coincident ductal carcinoma in situ are present, assessment of the extent and localization of both components is required for optimal therapeutic planning. We have used a mouse model of breast cancer to evaluate the feasibility of applying molecular imaging to assess the local status of cancers in vivo. Multi-tracer positron emission tomography (PET) and magnetic resonance imaging (MRI) characterize the transition from premalignancy to invasive carcinoma. PET tracers for glucose consumption, membrane synthesis, and neoangiogenesis in combination with a Gaussian mixture model-based analysis reveal image-derived thresholds to separate the different stages within the whole-lesion. Autoradiography, histology, and quantitative image analysis of immunohistochemistry further corroborate our in vivo findings. Finally, clinical data further support our conclusions and demonstrate translational potential. In summary, this preclinical model provides a platform for characterizing multistep tumor progression and provides proof of concept that supports the utilization of advanced protocols for PET/MRI in clinical breast cancer imaging.

## Introduction

Accurate identification of high- and low-risk neoplasms and monitoring their progression is currently one of the more challenging problems in breast cancer. Risk is assessed primarily by tumor stage (size and spread including metastases) and tumor grade (in breast, a standardized histologic score encompassing proliferation and morphology). Tumor grade and phenotypic heterogeneity as well as coincident invasive and in situ lesions require detailed study of the pathology for accurate reporting but this remains subjective. Our current classification schema fails to consider the indolent phenotype, leading to overdiagnosis and overtreatment^1^. Swanton described the phenomenon of intratumoral heterogeneity as a “process through time and space” in which alterations and mutations occur on different time scales in various regions of the same tumor^2^. Accurate comprehensive localized staging of breast lesions including the size and distribution of in situ and invasive areas of different grade/phenotype could be an important step in clinical management. The staging process is well defined and crucial for subsequent treatment decisions^3^. The current standard procedures for staging breast cancer include clinical examination, imaging by mammography and/or sonography, sometimes accompanied by magnetic resonance imaging (MRI), and histopathological analysis of biopsy and excision tissue specimens. Pathologists, limited to the local “snapshot” nature of a biopsy specimen, are well aware of tumor heterogeneity. They report the highest grade found after examining multiple tissue sections from any tumor biopsy^4^. Beyond biopsy-based staging and morphological imaging, molecular imaging modalities have the potential to noninvasively provide spatially resolved functional whole-lesion information. Positron emission tomography (PET) is established in daily practice in the fields of lung cancer diagnostics, prostate cancer diagnostics, or lymphoma^5–7^. Promising data have also been produced in breast cancer using molecular imaging to target glucose metabolism, proliferation, or receptor status^8–14^. A complete molecular characterization of tumor heterogeneity within single lesions can be important for clinical decision-making; however, PET measurements have not been standardized in breast cancer.

Many invasive ductal carcinomas (IDCs) also referred to as invasive mammary carcinomas of no special type (NST) are associated with components of ductal carcinoma in situ (DCIS). DCIS without an invasive component can sometimes progress over time to invasive carcinoma, but the time interval can be decades and in many cases progression may stall indefinitely. Some already consider the presence of a DCIS component within an invasive breast cancer a positive prognostic marker^15–17^. Therefore, differentiating between pure NST, DCIS, and mixed forms of NST/DCIS with noninvasive methods may be an important factor in clinical and preclinical evaluations. Like all clinical studies, validations of these results are necessarily dependent on the evaluation of large cohorts and statistical analysis.

In order to demonstrate the power of in vivo molecular imaging to characterize intralesional heterogeneity, we employed multiparametric PET/MRI to the transgenic polyomavirus middle T (FVB/N-Tg(MMTV-PyVT)634Mul/J) derived mammary intraepithelial neoplastic outgrowth (MIN-O) model^18^. This allowed the in vivo evaluation of heterogeneous cell populations during neoplastic progression.

The transplanted MIN-O grows contact-inhibited within the mouse mammary fat pad^19^. On histological examination, the MIN-Os do not form normal branching mammary trees, but form abnormal hyperplastic outgrowths^18^. The most peripheral growing edge forms modified terminal end buds that extend into the fat pad but lack the orderly organization of the normal terminal end bud^18^. The layer behind the growing edge contains more differentiated cells which typically form disorganized ducts with irregular alveoli. As the transplant grows, an inner core of ducts and alveolar structures extend. This zone is heterogenous and, broadly described, has more well-differentiated areas of glands with abundant eosinophilic cytoplasm and areas of differentiated hyperchromatic dysplastic alveolar cells. These hyperchromatic dysplastic cells are, CA-IX positive and could be considered “high nuclear grade” MIN lesions. Previous work employing the same MIN-O mouse model described the peripheral growing edge as “proliferation zone” and the encapsulated central region as “differentiation zone”, where the invasive adenocarcinoma develop^19^. Thus, alike the human disease, this model undergoes a histologically identifiable, multistep neoplastic progression to DCIS-like, premalignant MIN and invasive carcinoma (IC)^18,20–24^ (Suppl. Figure S1). In this study, PET biomarkers have been used to detect changes in tumor metabolism over time in the same animals to track the transition to malignancy. Our panel of biomarkers included [^18^F]fluoro-2-deoxy-2-D-glucose ([^18^F]FDG), [^11^C]choline ([^11^C]Chol), and quantified the expression of α_V_β_3_-integrin with [^68^Ga]Ga-NODAGA-c(RGDfK) ([^68^Ga]RGD)^25^. For each time point, autoradiography with subsequent staining of the slides co-registered PET tracer uptake with histopathology. Advanced analytic techniques for identifying intratumoral heterogeneity^26^ were applied to the data and provided unique insight into the dynamics of neoplastic progression. Our studies also included pre-lactating and lactating mammary gland controls to document normal physiological processes of increased metabolism and proliferation. Finally, exemplary patient data from a study on intratumoral heterogeneity in [^18^F]FDG PET/MRI of primary breast cancer patients were retrospectively analyzed to assess and illustrate the results in a clinical setting.

## Results

### Intratumoral staging using [^18^F]FDG-PET

To differentiate neoplastic stages during tumor development from premalignant DCIS-like MIN to IC, we investigated the glucose metabolism of lesions at different time points during tumorigenesis by using [^18^F]FDG PET (Suppl. Figure S2a). Four weeks after MIN-O transplantation (w4), hematoxylin and eosin (H&E) histology and whole-mount staining showed that the fat pads were filled with MIN tissue with no evidence of malignant tumors (Suppl. Figure S2b, c). Eight weeks post transplantation (w8), MIN and low amounts of invasive malignancy appeared in single lesions. However, by 11 weeks post transplantation (w11), tumors grew invasively through the border of the mammary fat pads (Suppl. Figure S2 b, c).

**Fig. 1:**
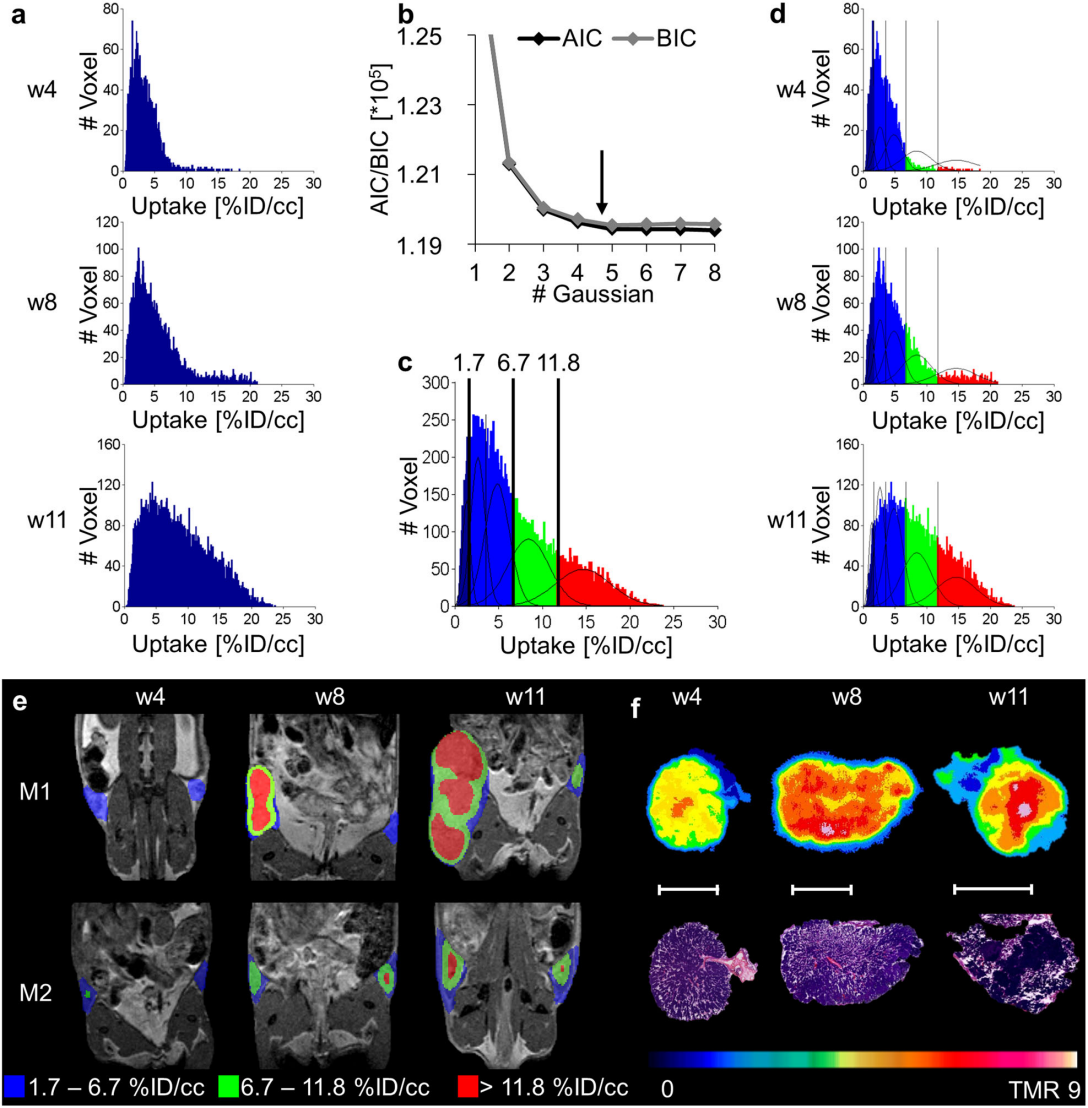
GMM analysis of [^18^F]FDG over time correlates with different tumor stages. **a** Summed histograms of all analyzed lesions of the single time points (w4: *n* = 16; w8: *n* = 15; w11: *n* = 13). Due to spillover effects from surrounding tissues (e.g., bladder), some lesions were excluded from the analysis. The three histograms were summed, and GMM analysis was performed on the summed data of all time points. **b** AIC and BIC for different numbers of Gaussian distributions, reaching its minimum at a sum of 5 Gaussian distributions. **c** Calculated thresholds from the 5 Gaussian mixture model were transferred to the summed histogram to separate the populations. **d** Applying the thresholds to the single time points demonstrates the appearance of the low uptake population in blue (< 6.7%ID/cc), the moderate uptake population in green (6.7–11.8%ID/cc), and the highest uptake population in red (> 11.8%ID/cc) for different time points. **e** Parametric maps of the lesions of two representative mice confirm the appearance of the increased uptake populations (green and red) during tumorigenesis. **f** Autoradiography of different tumor slides and corresponding H&E verified the highest [^18^F]FDG uptake in solid tumor regions, whereas hyperplastic regions of the lesions showed the lowest uptake. The white scale bar indicates 5 mm.

Mean and maximal value analyses were performed based on whole inguinal mammary fat pads. Both analyses revealed a trend towards higher [^18^F]FDG accumulation along the different steps of disease progression to IC and yielded high standard deviations (SDs) (Suppl. Figure S2d, e). Significant differences appeared in maximal value analysis between the time points (5.8 ± 2.1%ID/cc in w4; 9.6 ± 4.3%ID/cc in w8; 14.0 ± 5.8%ID/cc in w11, Suppl. Figure S2d, e). However, the PET images and ex vivo analysis revealed heterogeneous uptake within the individual lesions (Suppl. Figure S2a, Fig. 1).

Advanced, voxelwise analysis of summed [^18^F]FDG uptake in all lesions at each time point demonstrated a shift in voxel values over time towards higher uptake (Fig. 1a). As the summed histogram of all time points covered all uptake populations that appeared over the entire study duration, this summed dataset was fitted using a Gaussian Mixture Modell (GMM). The fitting criteria Akaike Information Criterion (AIC) and Bayesian Information Criterion (BIC) defined a sum of five Gaussian distributions as the best fit for the study (Fig. 1b, c). Applying the corresponding thresholds to the single time points demonstrated the appearance of the populations over time (Fig. 1d). The first population with uptake values < 1.7%ID/cc was identified as a peripheral background population. The following two populations with values of 1.7–3.6%ID/cc and 3.6–6.7%ID/cc could not be separated by in vivo imaging, as both populations already existed at the early time point w4 when only fat tissue and hyperplasia were present within the fat pads. Whether the fat corresponds to one population and the hyperplasia to another could not be verified due to the spatial resolution of the PET (1.6 mm maximal achievable resolution^27^). Therefore, these two populations were summed together as the fat and hyperplasia population in blue. The fourth population in green (6.7–11.8%ID/cc) appeared clearly in w8 when histological analysis identified DCIS-like MIN lesions (Suppl. Figure S2b). The remaining population in red with uptake values > 11.8%ID/cc emerged in a high amount at late time points in tumor development when the tumors showed invasive growth in a 3-dimensional manner (Fig. 1d & e, Suppl. Figure S2b). The presence of high-uptake populations (red and green) at earlier time points could be explained by single lesions that presented faster development and early invasiveness (mouse M1, Fig. 1e). Autoradiography results confirmed increasing uptake of [^18^F]FDG, from low (w4) and moderate uptake in premalignant regions (especially in w8) to the highest uptake in invasive carcinoma (especially in w11) (Fig. 1f).

### Metabolic characterization of tumorigenesis

#### Mean value analysis

**Fig. 2:**
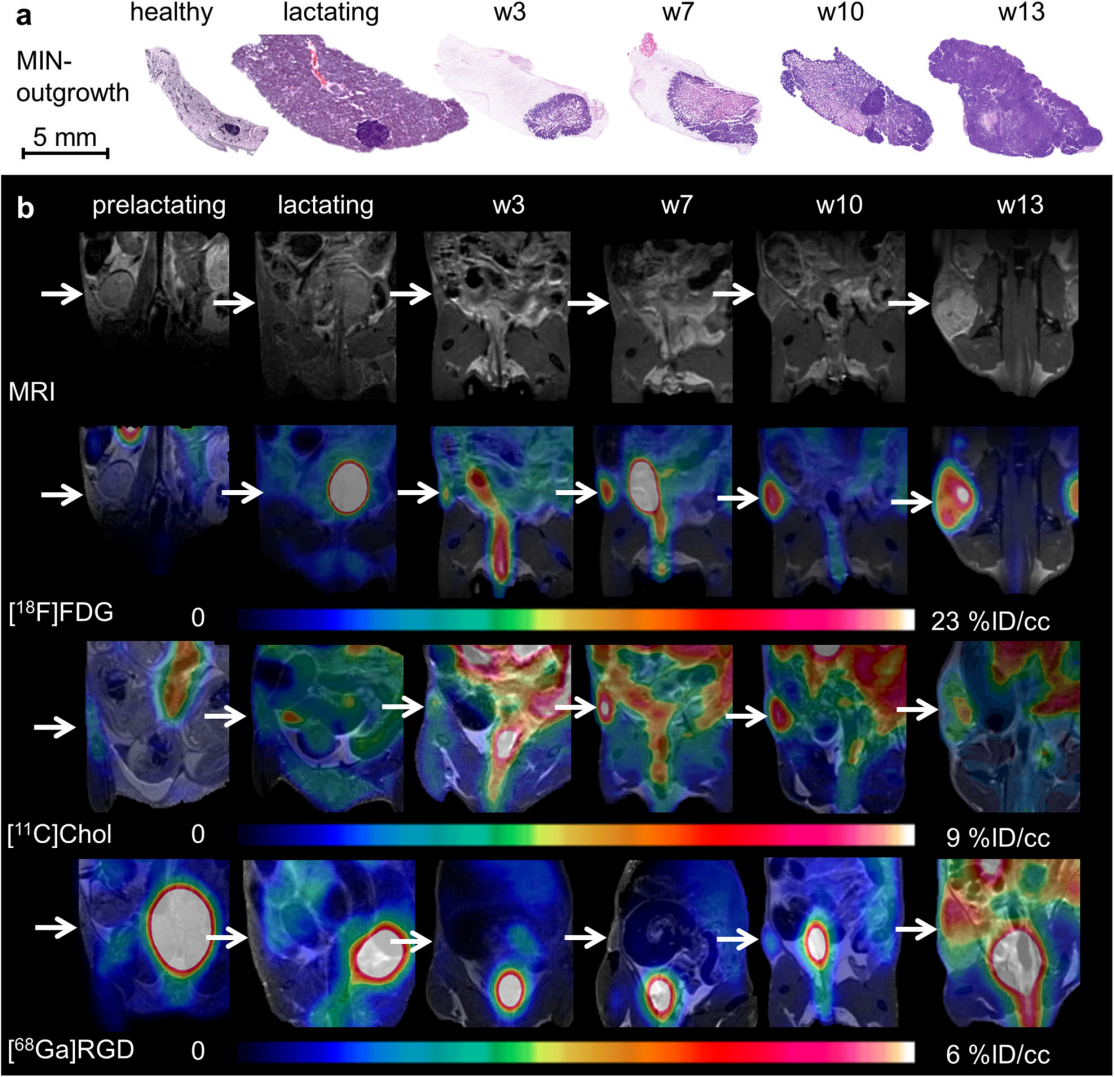
Tracer accumulation during tumorigenesis of the MIN-O model compared to prelactating and lactating mammary glands. **a** H&E histology slides illustrating the development of the MIN-O lesions within the mammary glands compared to controls of a healthy and lactating gland. **b** Anatomical MRI and fused PET/MR images of a representative MIN-O mouse over time compared to pre- and lactating glands for the investigated tracers [^18^F]FDG, [^11^C]Chol and [^68^Ga]RGD (white arrows indicate the region of the glands and MIN-O, respectively), and tracer uptake of all tracers increased during tumorigenesis over time.

Following the intratumoral staging approach using [^18^F]FDG, subsequent investigation addressed proliferation and angiogenesis during tumor development. Five mice were measured at the time points w3, w7, w10, and w13 with MRI, [^18^F]FDG, [^11^C]Chol, and [^68^Ga]RGD. Five additional mice were only measured with [^18^F]FDG for subsequent ex vivo analysis at each time point. Prelactating and lactating glands served as control tissue (Fig. 2).

H&E histology demonstrated tumor development in comparison to healthy, prelactating, and lactating mammary glands (Fig. 2a). The pre- and lactating glands, as well as the early lesion stages in w3, presented no or very low uptake of [^18^F]FDG, [^11^C]Chol, and [^68^Ga]RGD. In contrast, the latest stage (w13) exhibited high uptake with a clear heterogeneous pattern (Fig. 2b).

**Fig. 3:**
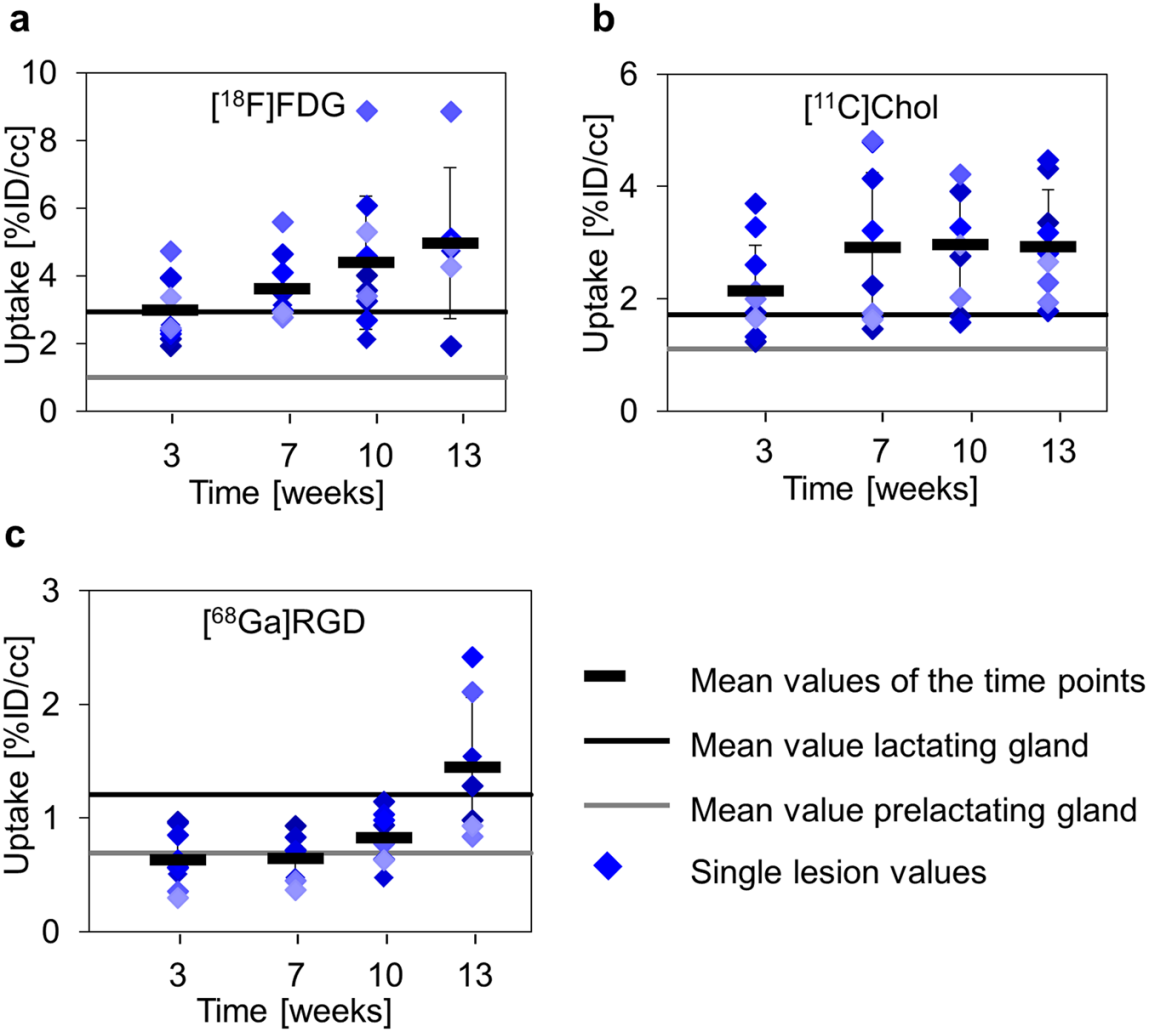
Mean value analysis of tracer uptake. Mean values (black bars) ± standard deviation, as well as single values, for every lesion (filled diamonds) of the investigated tracer were plotted over time and compared to prelactating (gray line) and lactating (black line) mammary glands. **a** [^18^F]FDG uptake showed a steady increase in uptake from w3, starting at the level of the lactating glands to w13. **b** [^11^C]Chol showed a slight increase from w3 to w7 and a stable mean value from w7 until w13. **c** [^68^Ga]RGD uptake was observed in the range of the prelactating glands from w3 to w10 and increased from w10 to w13. A statistically significant difference between MIN-O lesions and pre- or lactating glands was not observed for any of the tracers.

A mean value analysis of prelactating glands showed tracer uptake in the range of background accumulation (Table 1). However, lactating glands revealed increased uptake compared to background (Fig. 3a–c). During tumorigenesis, mean [^18^F]FDG uptake increased continuously (Fig. 3a, Table 1), while [^11^C]Chol uptake rose initially from w3 to w7 and remained stable thereafter (Fig. 3b, Table 1). [^68^Ga]RGD uptake surpassed background levels after only w10 (Fig. 3c, Table 1). None of the tracers reached significant differences in mean uptake values compared to lactating glands throughout the whole course of the study.

**Table 1:** Results of mean value analysis.

	[^18^F]FDG	[^11^C]Chol	[^68^Ga]RGD
Tissue	%ID/cc [MBq]	%ID/cc [MBq]	%ID/cc [MBq]
Muscle	1.0 ± 0.3	1.4 ± 0.4	0.5 ± 0.3
Pre-lactating glands	1.0 ± 0.1	1.3 ± 0.3	0.7 ± 0.2
Lactating glands	2.9 ± 0.2	1.7 ± 0.2	1.2 ± 0.7
Tumor (w3)	3.0 ± 0.9	2.1 ± 0.8	0.6 ± 0.2
Tumor (w7)	3.6 ± 0.9	2.9 ± 1.3	0.6 ± 0.2
Tumor (w10)	4.4 ± 2.0	3.0 ± 1.0	0.7 ± 0.4
Tumor (w13)	3.7 ± 3.0	2.9 ± 1.0	1.4 ± 0.6

#### Cluster analysis

[^18^F]FDG clustering was performed only in the first study. The second study data served as a test data set, where thresholds defining the cluster borders from the first study were applied, and confirmed previous clustering (Suppl. Figure S3). As a difference in both studies, tumor development was slower in the second study. However, the cluster analysis reflected this fact accurately, showing a slower progression from low and moderate to high uptake populations (Suppl. Figure S3c–f).

**Fig. 4:**
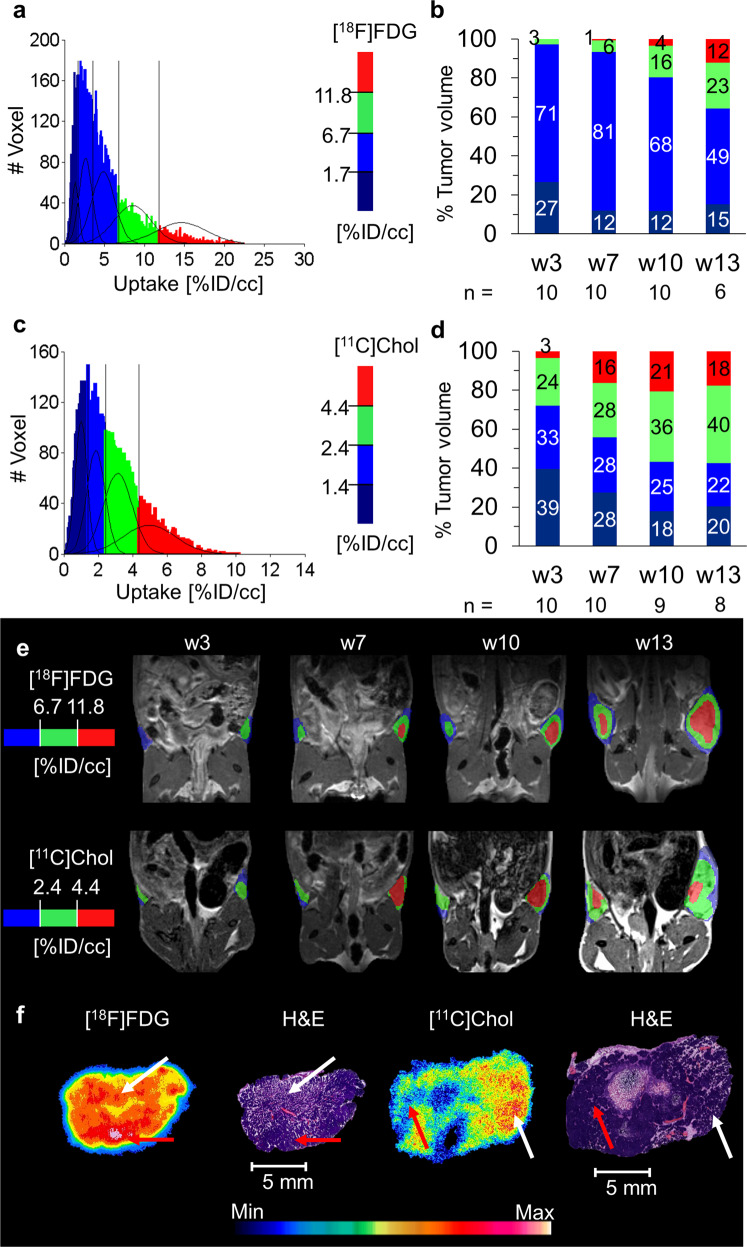
[^18^F]FDG and [^11^C]Chol clustering mismatch identifies tumor stages. **a** The summed and fitted histogram of [^18^F]FDG, as well as **b** the volumetric percentage of the uptake populations within the lesions for every time point, demonstrated a high amount of the low [^18^F]FDG uptake populations within the lesions in this study, including at later time points. The high uptake population (> 11.8%ID/cc) showed a slight increase over time up to 12% of the late-stage lesions in w13. **c** The summed histogram of all time points of [^11^C]Chol was analyzed as described for [^18^F]FDG, and the sum of four Gaussian distributions were identified as the best model. **d** The volumetric percentage of the uptake populations within the lesions for every time point demonstrated a lower amount of low [^11^C]Chol uptake populations compared to [^18^F]FDG. After a slight increase of the higher uptake populations (green, 2.4–4.4%ID/cc and red, > 4.4%ID/cc) from w3 to w7 and w10, both remained relatively stable until w13. **e** The parametric maps showed the appearance of the highest uptake populations at w7 in single tumors for both tracers, staying stable over time for [^11^C]Chol and increasing for [^18^F]FDG. **f** The autoradiography results of representative tumor slides demonstrated the highest [^18^F]FDG uptake in IC regions (red arrows), whereas the highest [^11^C]Chol uptake was observed in proliferating regions including DCIS-like high-grade MIN (white arrows) rather than invasive tumor regions.

Similar to [^18^F]FDG clustering (Fig. 4a, b), cluster analysis of [^11^C]Chol also resulted in a sum of 4 Gaussian distributions with thresholds of 1.4%ID/cc, 2.4 %ID/cc, and 4.4%ID/cc (Fig. 4c, d). The population with the highest [^11^C]Chol uptake (>4.4%ID/cc) increased from w3 (3% of the total tumor volume) to w7 (16% of the total tumor volume) and remained relatively stable until w13 (18% of total tumor volume) (Fig. 4d), correlating with the appearance of premalignant DCIS-like MIN regions (w7–w13) in the histological description of the MIN-O model (Fig. 2a). In contrast, the population with the highest [^18^F]FDG uptake (> 11.8%ID/cc) increased continuously from w3 (0% of total tumor volume) until w13 (12% of total tumor volume) (Fig. 4b). This increase in invasive tumor volume was also supported by histology, which also identified further subtypes in w13 (Suppl. Figure S4)^18^.

Both tracers, [^18^F]FDG and [^11^C]Chol, separated DCIS-like MIN and IC from the lactating glands using the corresponding population thresholds ([^18^F]FDG uptake > 6.7%ID/cc, [^11^C]Chol uptake > 4.4%ID/cc, Table 1).

Histological analysis supported these results and revealed changing histological patterns over time (Suppl. Figure S4): in w3, only the atypical hyperplastic type MIN tissue was observed. More pronounced higher-grade MIN appeared from w7 through w13. First signs of invasive growth were detectable in individual transplants as early as in w8 (study 1). In a second study, detection began in w10 (study 2), which involved the entire transplant by w13. Within these large metaplastic tumors, cystic, eosinophilic, and glandular differentiation patterns appeared (Suppl. Figure S4).

The specific uptake pattern of [^18^F]FDG and [^11^C]Chol within the same tumor differed (Fig. 4e). Autoradiography verified the highest [^18^F]FDG uptake in areas with invasive tumor growth within the tissue transplant sections, while the highest [^11^C]Chol uptake appears correlated with premalignant MIN regions (Fig. 4f).

**Fig. 5:**
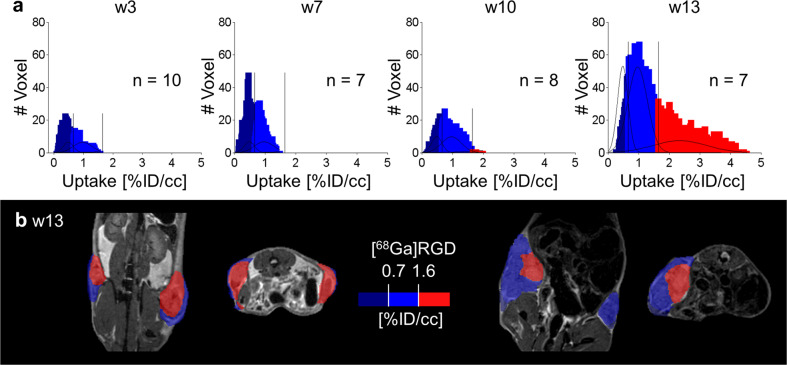
GMM analysis of [^68^Ga]RGD characterized IC regions. **a** The summed histogram of [^68^Ga]RGD uptake of all tumors and all time points was fitted and analyzed as described above for [^18^F]FDG, resulting in a sum of 3 Gaussian distributions as the best model. The thresholds 0.7 and 1.6%ID/cc were applied to the histograms of the single time points. **b** Representative PET/MR images revealed the highest uptake population (red, > 1.6%ID/cc) in only large tumors.

Clustering of [^68^Ga]RDG revealed a sum of only three uptake populations with thresholds of 0.7%ID/cc and 1.6%ID/cc (Fig. 5a). Subtracting the background uptake in muscle and lactating glands (Table 1), only the highest uptake population remained. This positive [^68^Ga]RGD population was only observed in large tumors (Fig. 5a, b).

### Immunohistochemical verification

**Fig. 6:**
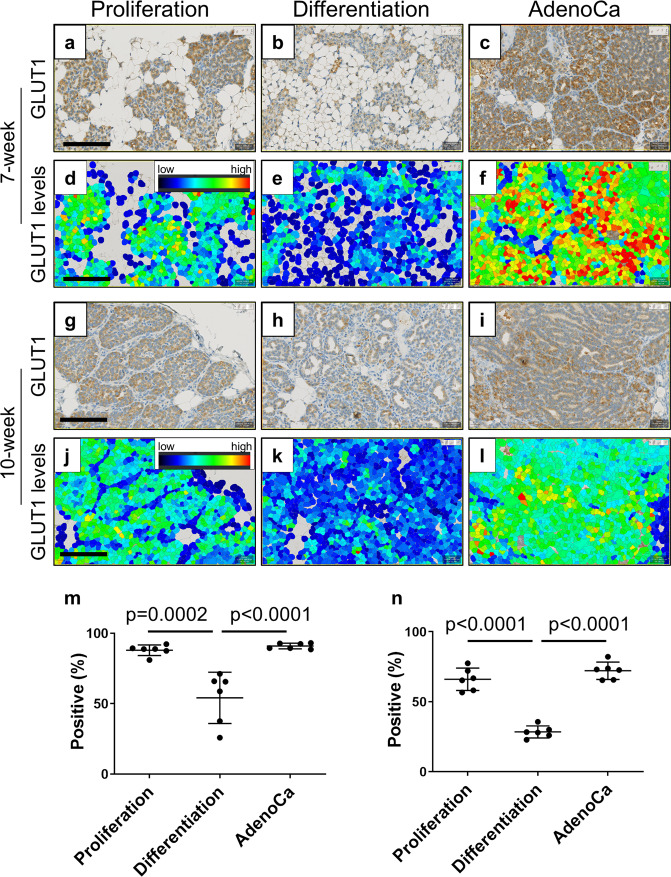
Quantitative GLUT1 expression. The higher levels of GLUT1 positive cells are observed at proliferation zones and tumor areas. GLUT1 stained MIN-O tissues at 7-week **a**–**f** and 10-week **g**–**l** were analyzed for positive cell density **m**, **n** and the levels of GLUT1 **d**–**f**, **j**–**l**. Images were analyzed at proliferation zones **a**, **d**, **g**, **j**, differentiation zones **b**, **e**, **h**, **k** and tumor areas (indicated as AdenoCa; **c**, **f**, **i**, **l**. The color indicates the levels of GLUT1 **d**–**f**, **j**–**l** as brighter color as higher GLUT1 levels and dark color as lower GLUT1 levels. **m**, **n** Graphs show the measurement of GLUT1 positive cell densities (positive percentage) at proliferation zones, differentiation zones and tumor areas in **m** 7-week and **n**10-week MIN-O tissues. Bars represent mean value ± standard deviation. Scale bar indicates 100 µm. All images were adjusted to same magnification.

**Fig. 7:**
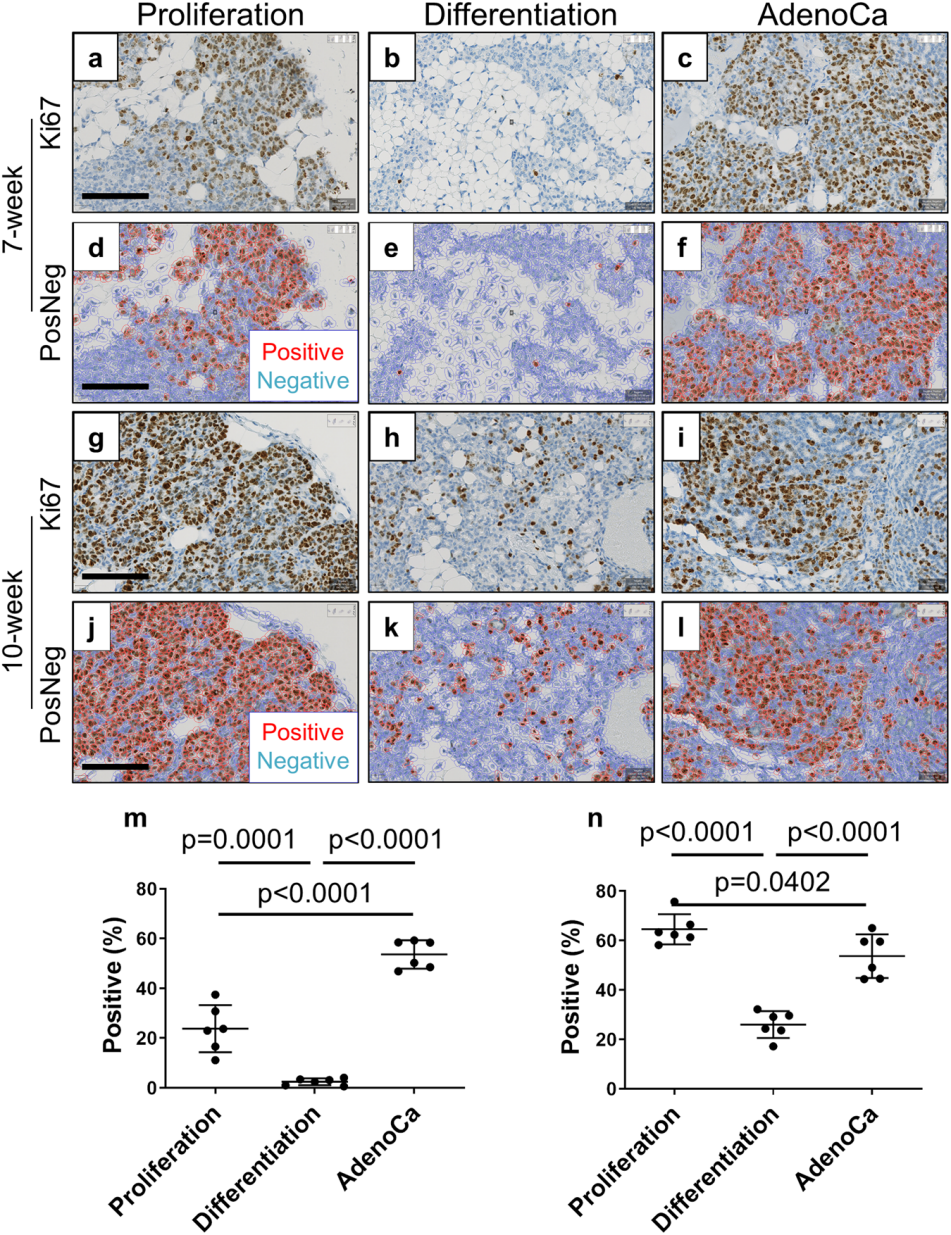
Quantitative Ki67 expression. Proliferation zones and tumor areas are highly proliferative compared with differentiation zones. Ki67 stained MIN-O tissues at 7-week **a**–**f** and 10-week **g**–**l** were analyzed for cell proliferation **m**, **n**. Images were analyzed at proliferation zone **a**, **d**, **g**, **j**, differentiation zones **b**, **e**, **h**, **k** and tumor areas (indicated as AdenoCa; **c**, **f**, **i**, **l**. The analysis for Ki67 positive cells were performed on QuPath by identifying nuclear Ki67 signals. Images shown as “PosNeg” indicate red cells as Ki67 positive and blue cells as Ki67 negative. The densities of Ki67 positive cells (positive percentage) were analyzed at proliferation zones, differentiation zones and tumor areas in **m** 7-week and **n** 10-week MIN-O tissues. Bars represent mean value ± standard deviation. **m** Scale bar indicates 100 µm. All images were adjusted to same magnification.

To correlate the in vivo imaging data to the biological characteristics of the tissues, immunohistochemical analyses of glucose transporter 1 (GLUT1) and proliferation (Ki67) were performed on 7-week and 10-week MIN-O tissues, which were further classified into proliferation zones, differentiation zones, and adenocarcinoma (Figs. 6, 7). Additional IHC included CD31- and β_3_-Integrin-staining for vessel density and neoangiogenesis, respectively (Suppl. Figure S5).

Regarding GLUT1 expression, more cells in the proliferation zones were positive than cells in differentiation zones at both 7 weeks (Fig. 6m; proliferation zones: Mean ± SD = 88.0 ± 3.8%; differentiation zones: Mean ± SD = 54.2 ± 18.2%, *p* = 0.0002, *n* = 6) and 10 weeks (Fig. 6n; proliferation zones: Mean ± SD = 66.1 ± 8.0%; differentiation zones: Mean ± SD = 28.4 ± 8.0%, *p* < 0.0001, *n* = 6). In addition, the levels of GLUT1 signal in proliferation zones’ positive cells were also stronger than that in differentiation zones (Fig. 6d, e, j, k).

The highest levels of GLUT1 signal were observed in adenocarcinomas (Fig. 6f, l) which also showed more positive cells than other zones on average (Fig. 6m, n; Mean ± SD = 91.0 ± 4.3% and 72.1 ± 6.2%, for 7-week and 10-week samples, respectively). The same trend was also observed in 13-week MIN-O tissues (data not shown).

This pattern of robust GLUT1 positivity, especially in adenocarcinoma, correlates greatly with the results derived from [^18^F]FDG uptake. Since the levels of GLUT1 are higher in adenocarcinoma compared to the proliferation and differentiation zones, this difference can be correlated with tumor progression.

In a similar fashion, Ki67 is also detected in a wide range of cells in 7-week and 10-week proliferation and differentiation zones (Fig. 7m, n; Mean ± SD = 23.8 ± 9.5% and 64.6 ± 6.1%, for 7-week and 10-week samples, respectively); whereas most cells in differentiation zones were negative for this biomarker (Fig. 7m, n; Mean ± SD = 2.4 ± 1.4% and 26.0 ± 5.4%, for 7-week and 10-week samples, respectively), constituting a significant difference between the two zones at both 7 weeks (*p* = 0.0001, *n* = 6) and 10 weeks (*p* < 0.0001, *n* = 6).

Notably, Ki67 expression remained consistently high in adenocarcinoma across these timepoints (Fig. 7m, n; Mean ± SD = 53.6 ± 5.7% and 53.7 ± 8.8%, for 7-week and 10-week samples, respectively).

Interestingly enough, a distinctive pattern was observed in β3-integrin expression where the positive cells’ signals appeared higher in adenocarcinoma while maintaining at relatively lower levels across all other zones (Suppl. Figure S5). In these adenocarcinoma areas, however, positive β3-integrin staining was not only limited to capillary veins but also greatly detected in tumor cells. Therefore, [^68^Ga]RGD uptake appeared to be mostly mediated by the tumor cells themselves.

Addressing blood vessel density and formation within the lesions, CD31 staining showed a dense but heterogenous distribution and dense blood vessel system for MIN regions and adenocarcinoma (Suppl. Figure S6). Our prior studies demonstrated the high level of vascularity in these lesions and the highly disorganized pattern^18^. A quantitative image analysis in MIN-O tissues was further impaired by potential vasculogenic mimicry^28^.

### Patient data

**Fig. 8:**
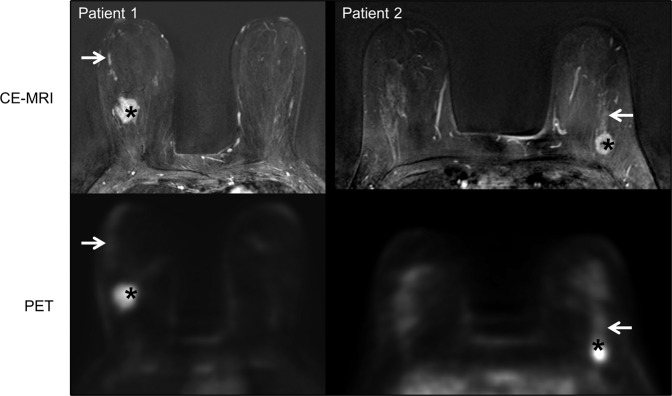
Representative images from patients. (Patient 1) A 48-year-old with biopsy-proven invasive ductal carcinoma (asterisks) in the right breast with extensive DCIS (arrows), which was proven in final histopathology. Axial subtraction image of contrast-enhanced dynamic breast MRI in the upper half shows the intensively enhancing lesion (asterisk) with a linear non-mass-enhancement (arrow) towards the nipple. In the lower half, axial PET shows the high SUV of the invasive tumor (asterisk; SUV mean 7.39) and low SUV in the localization of DCIS (arrow; SUV max 1.27). (Patient 2) A 46-year-old with biopsy-proven invasive ductal carcinoma (asterisks) in the left breast with associated DCIS (arrows), which was proven in final histopathology. Axial subtraction image of contrast-enhanced dynamic breast MRI in the upper half shows the intensively enhancing lesion (asterisk) with a linear non-mass-enhancement (arrow) towards the nipple. In the lower half, axial PET shows the high SUV of the invasive tumor (asterisk; SUV mean 7.39) and the low SUV in the localization of the DCIS (arrow; SUV max 1.27).

Histologically validated invasive tumor lesions of patients presented high [^18^F]FDG uptake with an SUV mean > 7. The highest uptake in DCIS regions (SUV max) was below 1.6, a threshold we defined in our previous work for less aggressive regions^26^ (Suppl. Table 1, Fig. 8).

## Discussion

This study was designed to demonstrate the potential of in vivo molecular imaging for determining the status of mammary neoplasias. Differentiation of pure NST, NST/DCIS, and DCIS without invasive carcinoma is essential for subsequent therapeutic decisions^29^. In the era of precision medicine and personalized treatment, this noninvasive approach should be applicable in human breast imaging and may provide an important new diagnostic approach especially in the setting of active surveillance of presumed pure DCIS^30^.

We demonstrate here, using a mouse model of DCIS with consistent progression to invasive carcinoma, the MIN-O transplant model^18^, that the precancer or MIN can be distinguished from invasive carcinoma using molecular PET/MR imaging and adequate analysis. Tumors in the MIN-O mouse model developed from experimentally defined precancerous cells within the transplanted MIN. The model has experimentally reproducible and morphologically distinct stages DCIS-like MIN and IC. Taken at a medium time point (e.g., 7w and 10w), each transplant has morphologically identifiable components of both MIN and invasive carcinoma. Thus, the model provides an experimentally verified and reproducible basis for the study of comparable human precancer progression to invasive breast cancer. These experiments represent biological proof of principle that can then be applied to the human disease.

Damonte et al. demonstrated that invasive tumor components develop from the “differentiation zone” comprised of premalignant low-grade more well-differentiated MIN^19^. Detection of this transition early in tumorigenesis could enable early preventive interventions. The in vivo detection and differentiation of these regions within early MIN tissues was limited by the spatial resolution of the PET scanner (1.6 mm maximal achievable resolution^27^) and, in this context, the partial volume effect. With the advent of next generation high-resolution, high-sensitivity PET scanners^31^, this restriction of PET imaging could be overcome in patients. In our study, the autoradiography (0.05 mm pixel length) proved increased [^18^F]FDG accumulation in the MIN-derived tumors compared to premalignant MIN. While not the only relevant glucose transporter, GLUT1 IHC further corroborates these findings. Furthermore, despite high levels of Ki67-expressing cells, invasive carcinoma appeared photopenic compared to MIN regions in [^11^C]Chol autoradiography. Thus, uptake mechanisms between MIN and IC might differ.

Due to the GMM-based voxelwise analysis, the PET imaging tracers [^18^F]FDG, [^11^C]Chol, and [^68^Ga]RGD demonstrated the neoplastic variations within each whole heterogeneous transplant lesion at single time points. Temporal changes were documented by repeated studies in the same animal providing a chronological map of tumor development. Each lesion could be subsequently co-registered and verified using autoradiography and histology. This noninvasive imaging approach decoded the intra-lesional pattern of the mammary neoplastic stages. In particular, [^18^F]FDG and [^11^C]Chol distinguished between premalignant MIN and IC, which was directly supported by autoradiography. These lesions also proved to differ from the physiologically increased metabolism and proliferation of prelactating and lactating mammary gland controls.

[^68^Ga]RGD-PET has been discussed as a promising imaging modality for the investigation of breast cancer, because the target of the RGD peptide, the activated α_V_β_3_-integrin, is overexpressed on newly generated blood vessels during neoangiogenesis^32^. α_V_β_3_-integrin has also been discussed as a potential therapeutic target^11,33^. In the MIN-O mouse model, in vivo imaging revealed increased [^68^Ga]RGD uptake only in late-stage invasively growing tumors, suggesting a late angiogenic switch during the development of invasive carcinoma. However β_3_-integrin IHC primarily showed overexpression on tumor cells of invasively growing tumors rather than on blood vessels. Therefore, this angiogenic switch could not be verified. In contrast, and supported by β_3_-integrin staining, accumulation of [^68^Ga]RGD was only observed in invasive tumors, making [^68^Ga]RGD a potentially important marker for IC-development in this mouse model.

The presence of activated α_V_β_3_-integrin on the tumor cells themselves, especially in breast cancer, is often associated with a metastatic phenotype^34–36^. This specific MIN-O line has a high incidence of lung metastasis at later time-points; α_V_β_3_-integrin expression could be an early step in metastasis^18^. However, examining the lungs of our mice at w13 did not reveal metastases. Nevertheless, the time course of α_V_β_3_-integrin expression on tumor cells and metastasis formation merits further study.

The timeline of occurrence of invasive carcinoma and associated DCIS is controversial. Mathematical modeling of DCIS and associated invasive carcinoma origins and development based on empirical marker analyses suggest that they arise simultaneously and grow in parallel, as opposed to the prior assumption that DCIS expands first, and a subsequent focus of invasion evolves as a subclone of the DCIS^37,38^. Intriguingly this is supported by single-cell sequence analysis of DCIS and adjacent invasive cancers which display identical subclones in both compartments^39^. In all its stages, the MIN-O model supports the parallel pathway theory for this model. That is, all information for the development of the malignant disease is already encoded in the MIN stages^18–21,38,40^.

A direct application of the MIN-O model to clinical studies remains debatable. [^18^F]FDG detected the more aggressive IC regions as compared to regions of MIN and normal tissue growth. This agreed with both our clinical results and the literature^16^. The additional information of higher [^11^C]Chol uptake at the proliferation zone and DCIS-like high-grade MIN could translate into verification of DCIS in the background of persistent breast tissue. This however could not be verified because the clinical data were not available for our patients. Further, Contractor et al. reported [^11^C]Chol uptake in ER-positive breast cancer^41^. Clearly, differences in tracer uptake and the underlying physiological processes in choline and glucose metabolism need to be further investigated.

Characterization of the tumorigenesis of mammary cancer originating from transplanted atypical hyperplastic outgrowths was proven to be feasible using the radiotracers [^11^C]Chol, [^18^F]FDG, and [^68^Ga]RGD. Advanced imaging analysis distinguished intra-transplant regions of premalignant DCIS-like high-grade MIN from IC by [^11^C]Chol and [^18^F]FDG. Supported by β_3_- integrin IHC, [^68^Ga]RGD-PET imaging indicated the progression from MIN to adenocarcinoma, suggesting further correlations with a metastatic breast cancer phenotype. Our results also support the hypothesis of parallel development of IC and DCIS in this tumor model. Most importantly, our study shows that molecular imaging enables a localized differentiation between premalignant disease and invasive carcinoma. Further application of preclinical therapeutic studies and translation of the imaging protocols to clinical investigations can provide a better understanding of DCIS development and provide an approach that reduces overtreatment. We hope that the lessons learned from our in vivo experiments with a model system of cancer progression will guide and stimulate similar studies of human breast cancer.

## Methods

### Mice

All experiments were performed on female FVB/N mice (Charles River Laboratories, Sulzfeld, Germany). The MIN-O line was generated by transplantation of MIN lesions to create transplantable outgrowth lines (MIN-O) in the mammary glands of FVB/N-Tg(MMTV-PyVT)634Mul/J mice at the University of California, Davis, USA. Frozen MIN-O (line D) tissue was transferred to our lab for implantation into gland-cleared mammary fat pads of FVB/N mice and subsequent serial transplantation. Both inguinal mammary fat pads of the animals were cleared from the developing gland structure, and tissue was transplanted on both sides when mice were 3 weeks old, as previously described^18,42^. All animals were housed under standardized environmental conditions (22 ± 2 °C room temperature, 55 ± 10% relative humidity, and 12 h light-dark phases) with free access to food and water. Mice developed palpable tumors at 13–16 weeks of age (10–13 weeks post transplantation) in the transplanted mammary glands. Tumor development was monitored from week 3 through week 13 post transplantation.

### Preclinical in vivo imaging

In vivo studies were performed in a sequential PET/MRI setup using a dedicated 7 T small-animal MR scanner (ClinScan, Bruker BioSpin GmbH, Ettlingen, Germany) and a small-animal PET scanner (Inveon dedicated PET, Siemens Healthcare, Knoxville, TN, USA)^27,43^. [^18^F]FDG was synthesized according to our marketing license, [^11^C]Chol and [^68^Ga]RGD followed our published procedures^25,44^. Animals were anesthetized with 1–2% isoflurane evaporated in breathing air. PET tracers were intravenously injected. After 10 min PET emission scan, a 13 min PET transmission scan for attenuation correction was performed. Subsequently, the animals were transferred on the same bed to the MRI scanner. Three-dimensional T2-weighted turbo spin-echo MRI (TSE; TR 2500 ms, TE 202 ms, voxel size 0.27 × 0.27 × 0.27 mm^3^) provided anatomical references. Table 2 summarizes relevant study details.

**Table 2:** Tracer uptake times and injected doses.

Tracer	IA	Uptake time	TP	PET EM	PET TX	MRI
	[MBq]	[min]	[weeks]	[min]		Anatomy
S1: [^18^F]FDG	13 ± 2	66 ± 3	4,8,11	10	Yes	Yes
S2. [^18^F]FDG	13 ± 1	60	3,7,10,13	10	Yes	Yes
S2: [^11^C]Chol	13 ± 1	40	3,7,10,13	10	Yes	Yes
S2: [^68^Ga]RGD	13 ± 1	70	3,7,10,13	10	Yes	Yes

*S1* Metabolic alterations during tumor development, *S2* Multiparametric characterization of lesion stages compared to the lactating gland, *IA* injected activity, *TP* time points, *EM* emission, *TX* transmission. Data are displayed as mean ± standard deviation, where applicable.

All experiments were performed according to animal use and care protocols approved by local authorities (regional council Tübingen).

#### Metabolic alterations during tumor development

Tissue metabolism during tumor development (*n* = 10 mice, bearing 20 transplanted lesions) was investigated using the combined PET/MRI protocol 4, 8, and 11 weeks after transplantation (w4, w8, and w11). As one mouse was sacrificed in w4 and 2 mice in w8 for ex vivo analyses, 9 and 7 mice were measured in w8 and w11, respectively. The remaining 7 mice were sacrificed after the last measurement in w11, and lesions were excised for ex vivo analyses.

#### Multiparametric characterization of lesion stages compared to the lactating gland

To account for an additional measurement time point and tumor growth, the imaging time points were adjusted. Five animals in weeks 3, 7, and 10 post transplantation (w3, w7, and w10) and 4 animals in week 13 post transplantation (w13) were measured on temperature-controlled animal beds. All measurements were performed in a sequential PET/MRI setup after intravenous injection of 13 ± 1 MBq of [^18^F]FDG, [^11^C]Chol, or [^68^Ga]RGD (Radiopharmacy, University Hospital Tuebingen, Tuebingen, Germany). All tracers were measured in w3, w7, w10, and w13 within one week. Of note, [^11^C]Methionine and [^18^F]FMISO have also been measured but did not provide further information.

An additional five mice were measured only with [^18^F]FDG-PET/MRI. Two of these five mice were sacrificed in w3 and w7 and one in w10, and lesions were excised for ex vivo analysis. One additional mouse from the multi-tracer measurements was sacrificed in week 10 for ex vivo analysis, resulting in 2 mice per time point for ex vivo analyses. One mouse died during w13 before [^68^Ga]RGD examination; therefore, an additional mouse from the same transplantation was measured only with [^68^Ga]RGD during this week. All mice were sacrificed in w13, and lesions were excised for ex vivo analyses.

Furthermore, an additional six non-tumor-bearing mice (two for each tracer) were measured on day 16 ± 1 of their pregnancy and day 5 ± 1 of lactation to assess pre- and lactating mammary glands. Following the last measurement, one mouse per tracer was sacrificed, and the lactating mammary gland was excised for ex vivo analyses. The remaining mouse nursed the offspring of both mice.

### Ex vivo methods

Following the last in vivo measurement, mice were sacrificed, and lesions were excised and prepared for whole-mount staining, histology, and or autoradiography. For data correlation with [^18^F]FDG autoradiography, performed during the study time points, an additional two mice were injected with 13 ± 1 MBq [^11^C]Chol in w8 to perform an autoradiography experiment after 60 min of uptake.

#### Autoradiography

For autoradiography, tumors were embedded in Tissue-Tek O.C.T. compound (Sakura Finetek, Torrance, CA, USA) and frozen at −20 °C. Every 200 μm, a 20 μm section was cut with a cryostat (Leica Microsystems, Wetzlar, Germany) at −19 °C. A storage phosphor screen was placed on the slices and read out after an exposure time of 10 half-lives of the respective tracer with a pixel size of 50 μm using a STORM Phosphor-Imager (Molecular Dynamics, Sunnyvale, CA, USA). Tissue slices were then stained with hematoxylin and eosin (H&E), and whole-slide images were digitized using a digital slide scanner (NanoZoomer-XR C12000, Hamamatsu Photonics K.K., Hamamatsu-City, Japan). For normalization, autoradiography was analyzed as tumor-to-muscle-ratios (TMR), dividing the whole autoradiography plate of each mouse by the mean value of the muscle samples on the plate (ImageJ; National Institute of Health, Bethesda, USA^45^).

#### Histology

For histology, tumors were fixed in 4.5% formalin (SAV LP GmbH, Flintsbach am Inn, Germany) or zinc (IHC Zinc-Fixative, BD Biosciences, Franklin Lakes, USA) and embedded in paraffin (Paraplast® Embedding Media, McCormick Scientific, Leica Microsystems, Wetzlar, Germany). Formalin-fixed sections were cut at 4 μm, deparaffinized and stained with either H&E or with the respective antibodies for immunohistochemistry. Slides were stained using an automatic immunostainer (Discovery XT, Ventana Medical Systems, Inc., Tucson, USA) according to the manufacturer’s standard protocols with antibodies against CD31 (Abcam Inc., Cambridge, USA), β_3_-Integrin (Abcam Inc.), glucose transporter 1 (GLUT1, Abcam Inc.) and Ki67 (ThermoFisher Scientific, Waltham, USA). Images were acquired under a microscope (Axio Imager A1, Carl Zeiss AG, Oberkochen, Germany) using a coupled digital camera (ProgRes® C10plus, JenOptik, Jena, Germany) and the software ImageAccess (Version 6, Imagic Bildverarbeitungs AG, Glattbrugg, Switzerland) or extracted from digitized whole slide imaging (Nanozoomer, Hamamatsu) using the corresponding software.

### Data analysis

Preclinical PET data were reconstructed using the ordered-subsets expectation maximization algorithm with 3D post reconstruction (OSEM3D) (Inveon Acquisition Workplace, Siemens Healthcare). For in vivo data analysis, Inveon Research Workplace (IRW, Siemens Healthcare) was used. Corresponding PET and MR images were fused, volumes of interest (VOIs) covering the entire mammary fat pads were drawn based on the anatomical MRI data and the mean and maximal uptake values (%ID/cc) of the detected lesions were calculated.

Beyond this analysis, data were analyzed using a voxel-based analysis approach as previously described^26^. Briefly, histograms of the voxel values of PET data were calculated for the sum of all lesions within each time point and for the sum of all time points within the study. A Gaussian mixture model (GMM) was applied to cluster multiple uptake populations of the tracers over the course of the study during tumor development using Akaike information criterion (AIC) and Bayesian information criterion (BIC) to select the number of clusters (MATLAB, MathWorks, Natick, USA). The intersection between adjacent clusters defined the respective cluster boundaries.

Quantitative image analysis was performed digitized slides using the QuPath software (Version 0.2.3^46^). The analysis for GLUT1 positive cells was performed by identifying its signals in cytoplasmic/membrane. Images are shown as “GLUT1 levels” indicate the levels of GLUT1 as a heatmap with jet color scale. The GLUT1 positive cells (positive percentage) were analyzed at growth zones, differentiation zones and tumor areas in (m)7-week and (n)10-week MIN-O tissues. The analysis for Ki67 positive cells were performed by identifying nuclear Ki67 signals. Images shown as “PosNeg” indicate red cells as Ki67 positive and blue cells as Ki67 negative. The densities of Ki67 positive cells (positive percentage) were analyzed at growth zones, differentiation zones and tumor areas in (m)7-week and (n)10-week MIN-O tissues.

β_3_-Integrin staining was visualized similar to GLUT1. A quantitative evaluation of the different zones was omitted due to the positive staining of the adenocarcinoma cells.

### Patient data

Patient data were obtained as part of an IIT trial (German Clinical Trials Register, DRKS00013891) after approval by the responsible Ethics Committee of the Medical Faculty of the Eberhard-Karls-University and the University Hospital Tübingen and after patients provided written informed consent. Data were retrospectively analyzed to show two examples of imaging characteristics in tumors with extensive intraductal components of an invasive tumor. The patients presented with grade 3 unifocal and multifocal invasive breast cancer of no special type with associated high-grade DCIS, ER/PR neg, both IRS 0%, and lymph-node involvement. The two breast cancer patients received multimodal PET/MR, including 244 and 247 MBq of [^18^F]FDG and 0.1 mmol/kg Gd-based contrast agent (6 and 8 ml gadubutrol, respectively). To determine tracer uptake, ROIs were drawn on manually identified lesions. After surgical resection of the lesions, the tissue was histologically characterized.

### Statistical analysis

Mean and maximal value analysis was performed based on the uptake in %ID/cc (mean/max value ± standard deviation (SD)). For statistical analysis of PET data to compare the tracer uptake of different time points, Tukey-Kramer tests were performed (JMP, SAS Institute, Cary, USA). To compare tracer uptake of the time points in tumor development with the control tissue of pre- and lactating mammary glands, Dunnett’s tests, including Bonferroni correction, were performed (JMP).

Statistical comparison of quantitative image analysis was performed using a one-way ANOVA with Tukey-correction for multiple comparisons (GraphPad Prism V6).

### Reporting summary

Further information on research design is available in the Nature Research Reporting Summary linked to this article.

## Supplementary information

reporting summary

Supplemental Material

## Data Availability

All relevant data are presented in the manuscript and supplemental material. For subsequent data re-use a Data Usage and Access Committee (DUAC) will provide access to research-relevant data (anonymized) for research purposes after submitting a reasonable data usage request to the corresponding author.

## Code availability

Matlab scripts are available after submitting a reasonable code usage request to the corresponding author.
