# Supplementary material for: Intratumoral in vivo staging of breast cancer by multi-tracer PET and advanced analysis
Source: NPJ Breast Cancer. 2022 Mar 24;8:41. doi: 10.1038/s41523-022-00398-x (PMC8948294; doi:10.1038/s41523-022-00398-x)
Supplement: Supplementary file 1 — reporting summary [file 41523_2022_398_MOESM1_ESM.pdf]

nature portfolio

Corresponding author(s): Andreas Schmid

Last updated by author(s): Oct 27, 2021

Reporting Summary

Nature Portfolio wishes to improve the reproducibility of the work that we publish. This form provides structure for consistency and transparency in reporting. For further information on Nature Portfolio policies, see our Editorial Policies and the Editorial Policy Checklist.

Statistics

For all statistical analyses, confirm that the following items are present in the figure legend, table legend, main text, or Methods section.

n/a	Confirmed	
☐	☒	The exact sample size (n) for each experimental group/condition, given as a discrete number and unit of measurement
☐	☒	A statement on whether measurements were taken from distinct samples or whether the same sample was measured repeatedly
☐	☒	The statistical test(s) used AND whether they are one- or two-sided<br>*Only common tests should be described solely by name; describe more complex techniques in the Methods section.*
☒	☐	A description of all covariates tested
☒	☐	A description of any assumptions or corrections, such as tests of normality and adjustment for multiple comparisons
☐	☒	A full description of the statistical parameters including central tendency (e.g. means) or other basic estimates (e.g. regression coefficient) AND variation (e.g. standard deviation) or associated estimates of uncertainty (e.g. confidence intervals)
☒	☐	For null hypothesis testing, the test statistic (e.g. F, t, r) with confidence intervals, effect sizes, degrees of freedom and P value noted<br>*Give P values as exact values whenever suitable.*
☒	☐	For Bayesian analysis, information on the choice of priors and Markov chain Monte Carlo settings
☒	☐	For hierarchical and complex designs, identification of the appropriate level for tests and full reporting of outcomes
☒	☐	Estimates of effect sizes (e.g. Cohen's d, Pearson's r), indicating how they were calculated

Our web collection on statistics for biologists contains articles on many of the points above.

Software and code

Policy information about availability of computer code

Data collection	Preclinical PET and MRI Data were acquired and reconstructed with Inveon Acquisition Workplace, Version 01.950 and Siemens Syngo MR B15. Images were fused and data was extracted using Inveon Research Workplace.
Data analysis	PET-voxelvalues of VOI of the animals were analyzed using previsouly published Matlab Code. The code histograms the voxel values and fits gaussian distrubtions to the data. Intersections between the gaussians were defined as thresholds.

For manuscripts utilizing custom algorithms or software that are central to the research but not yet described in published literature, software must be made available to editors and reviewers. We strongly encourage code deposition in a community repository (e.g. GitHub). See the Nature Portfolio guidelines for submitting code & software for further information.

Data

Policy information about availability of data

All manuscripts must include a data availability statement. This statement should provide the following information, where applicable:

- Accession codes, unique identifiers, or web links for publicly available datasets
- A description of any restrictions on data availability
- For clinical datasets or third party data, please ensure that the statement adheres to our policy

All relevant data are presented in the manuscript and supplemental material. For subsequent data re-use a Data Usage and Access Committee (DUAC) will provide access to research-relevant data (anonymized) for research purposes after submitting a reasonable data usage request.

Field-specific reporting

Please select the one below that is the best fit for your research. If you are not sure, read the appropriate sections before making your selection.

☒ Life sciences ☐ Behavioural & social sciences ☐ Ecological, evolutionary & environmental sciences

For a reference copy of the document with all sections, see nature.com/documents/nr-reporting-summary-flat.pdf

Life sciences study design

All studies must disclose on these points even when the disclosure is negative.

Sample size	Based on previous experience, clustering of 20 tumors yielded a reliable training set for our analysis protocol. Subsqeuently, for the proof-of-concept study, 5 animals per group were approved for multi-tracer characerization study by the regional council.
Data exclusions	All available tumors were included in the study. However, as indicated in the manuscript, [11C]Methionine and [18F]FMISO PET were excluded from the report as these data did not further contribute.
Replication	All mice carried two tumors, one on either flank. MIN-O tissue was continously transplanted to maintain the MIN-O line throughout the duration of the experiment. No further replications were performed.
Randomization	Tumor development was monitored over time. Initial randomization was not relevant to the study.
Blinding	Blinding was not relevant to the study.

Reporting for specific materials, systems and methods

We require information from authors about some types of materials, experimental systems and methods used in many studies. Here, indicate whether each material, system or method listed is relevant to your study. If you are not sure if a list item applies to your research, read the appropriate section before selecting a response.

Materials & experimental systems

n/a	Involved in the study	
☐	☒	Antibodies
☒	☐	Eukaryotic cell lines
☒	☐	Palaeontology and archaeology
☐	☒	Animals and other organisms
☒	☐	Human research participants
☐	☒	Clinical data
☒	☐	Dual use research of concern

Methods

n/a	Involved in the study	
☒	☐	ChIP-seq
☒	☐	Flow cytometry
☒	☐	MRI-based neuroimaging

Antibodies

Antibodies used	Glut-1, ab652, abcam;<br>Integrin-b3, ab75872, abcam;<br>CD31, ab28364, abcam;<br>Ki67, Ki681R06, DCS;<br>HIF1a, ab8366, abcam
Validation	For validation purposes, the antibodies are evaluated on tissues representing relevant positive and negative controls during the establishing process.

Animals and other organisms

Policy information about studies involving animals; ARRIVE guidelines recommended for reporting animal research

Laboratory animals	female FVB/N mice (Charles River Laboratories, Sulzfeld, Germany), 2 - 16 weeks old
Wild animals	The study did not involve wild animals.
Field-collected samples	The study did not involve samples collected in the field.
Ethics oversight	All animal experiments were approved by the local authorities, Regional Council Tübingen, Germany.

Note that full information on the approval of the study protocol must also be provided in the manuscript.

Clinical data

Policy information about clinical studies

All manuscripts should comply with the ICMJE guidelines for publication of clinical research and a completed CONSORT checklist must be included with all submissions.

Clinical trial registration	German Clinical Trials Register, DRKS00013891
Study protocol	Only representative PET data were evaluated for this manuscript. The full study protocol is not relevant for this study.
Data collection	Two representative patients from the study were analyzed, focusing on FDG-PET data. Recruitment started April 23rd, 2018.
Outcomes	For this manuscript, two patients with DCIS/IC were selected.
