# Supplementary material for: Intratumoral in vivo staging of breast cancer by multi-tracer PET and advanced analysis
Source: NPJ Breast Cancer. 2022 Mar 24;8:41. doi: 10.1038/s41523-022-00398-x (PMC8948294; doi:10.1038/s41523-022-00398-x)
Supplement: Supplementary file 2 — Supplemental Material [file 41523_2022_398_MOESM2_ESM.pdf]

Supplemental Fig. S1

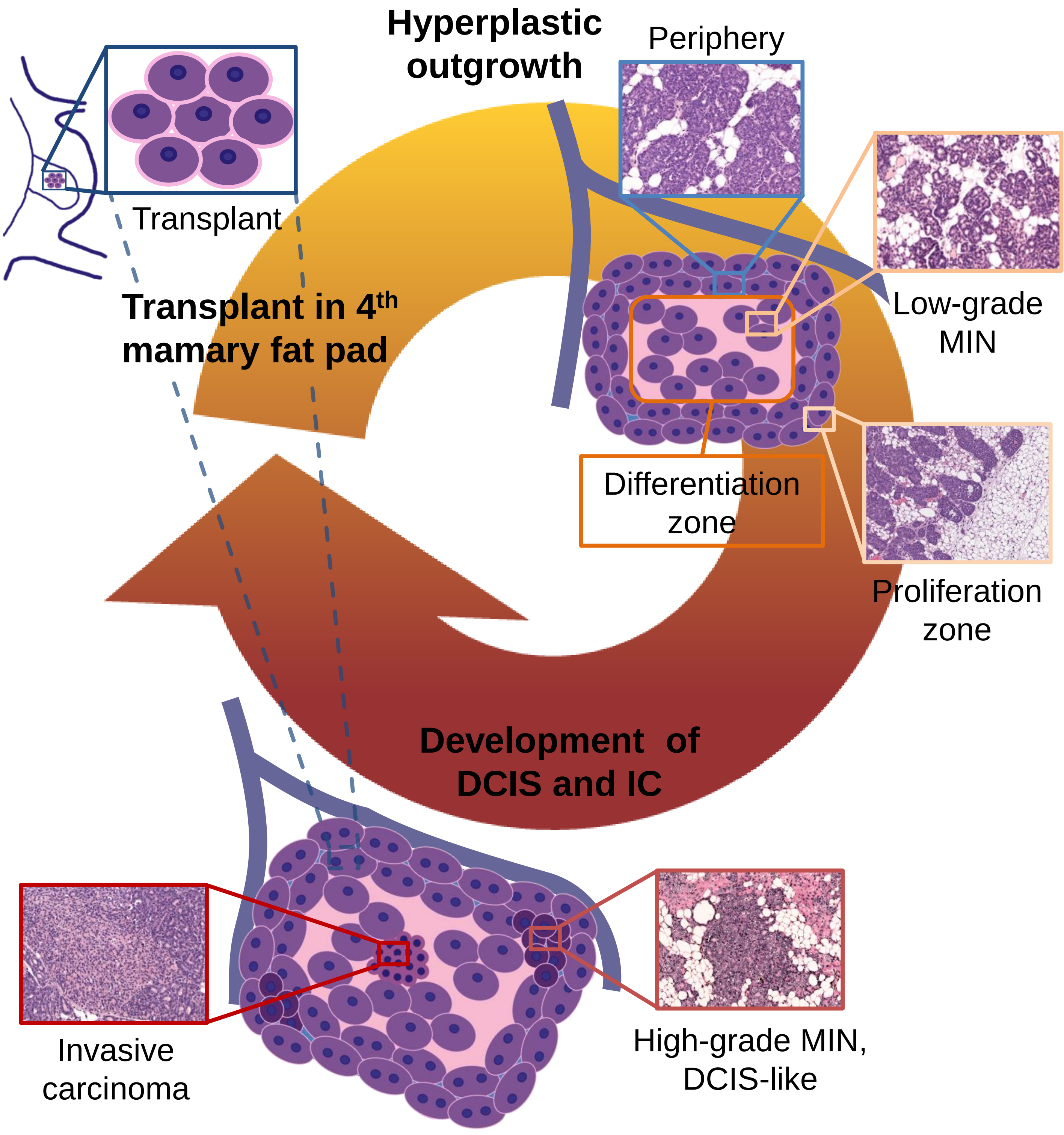

Supplemental Figure S1 : Schematic description of the MIN-O mouse model. A biopsy from the periphery of the MIN-O (fresh or cryopreserved), transplanted into the gland-cleared mammary fat pad #4, grows as an atypical hyperplasia within the boundaries of the mammary fat pad expanding via a terminal end bud-like peripheral proliferation zone. In the central part of the transplant, a differentiation zone develops as hyperplasia or low-grade MIN with normal spacing and contact inhibition. Over time, increasing numbers of high-grade MIN clusters may develop. Invasive carcinomas primarily arise, with time, in the central differentiation zone. Tissue from the peripheral proliferation zone serves as donor tissue for subsequent serial transplantations.

Supplemental Fig. S2

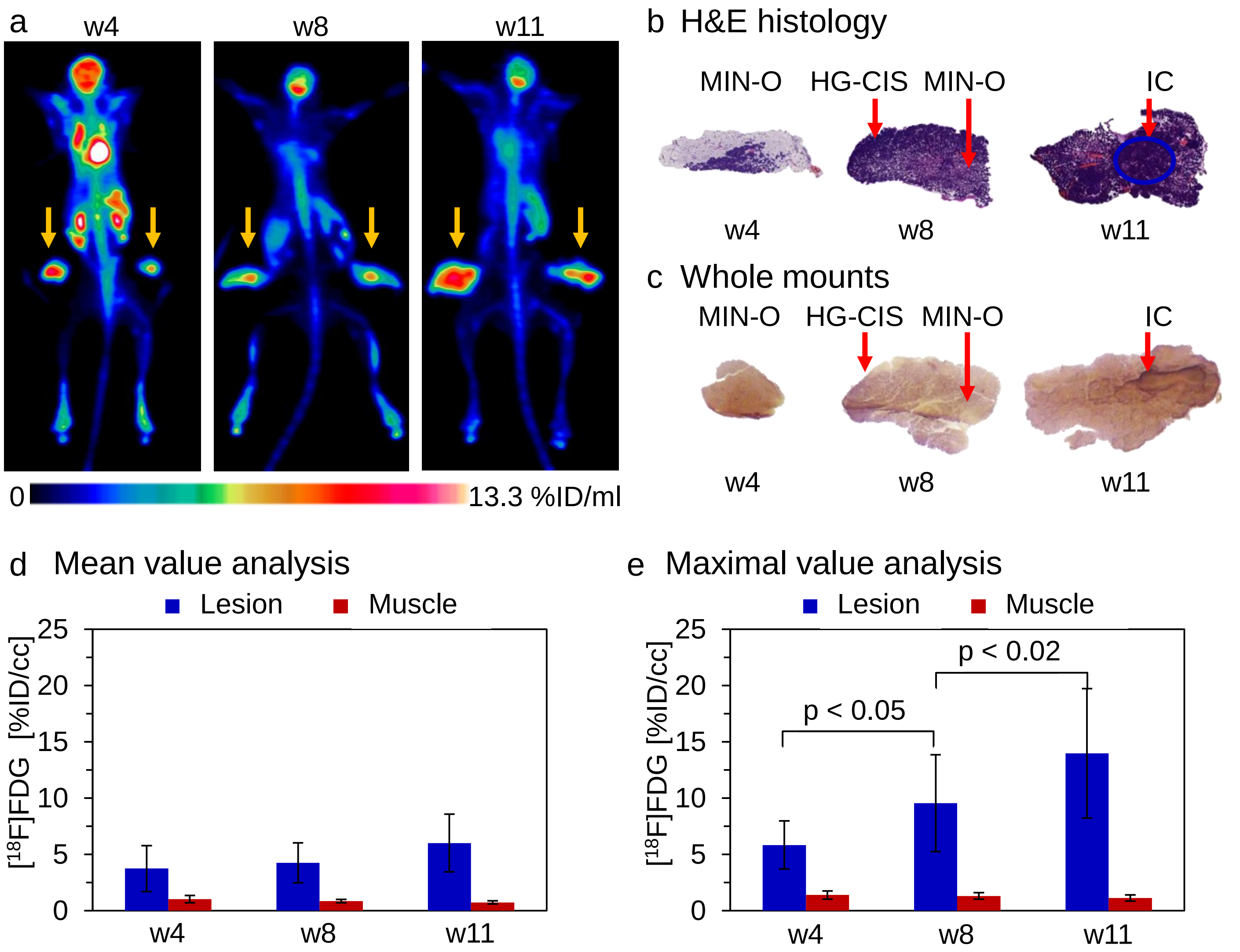

Supplemental Figure S2: Heterogeneity of the MIN-O model: (a) Heterogeneous *in vivo* [^{18}F]FDG uptake within MIN-O lesions over time (yellow arrows). (b) H&E histology of the heterogeneous structure of MIN-O tissue showing the stages of MIN outgrowth, foci of higher grade DCIS-like MIN (HG-CIS), and IC over time (red arrows). (c) "Whole-mounts" of MIN-O tissue showing the stages of hyperplasia, DCIS-like MIN and IC over time (red arrows). (d) Mean value analysis (%ID/cc ± SD) of the lesions over time showed higher [^{18}F]FDG uptake compared to muscle uptake for all time points but no significant increase over time. (e) Maximal value analysis (%ID/cc ± SD) further showed significantly increased [^{18}F]FDG uptake over time. Statistical analysis was performed using Tukey-Kramer tests for multiple group comparisons. Error bars indicate the standard deviation (SD).

Supplemental Fig. S3

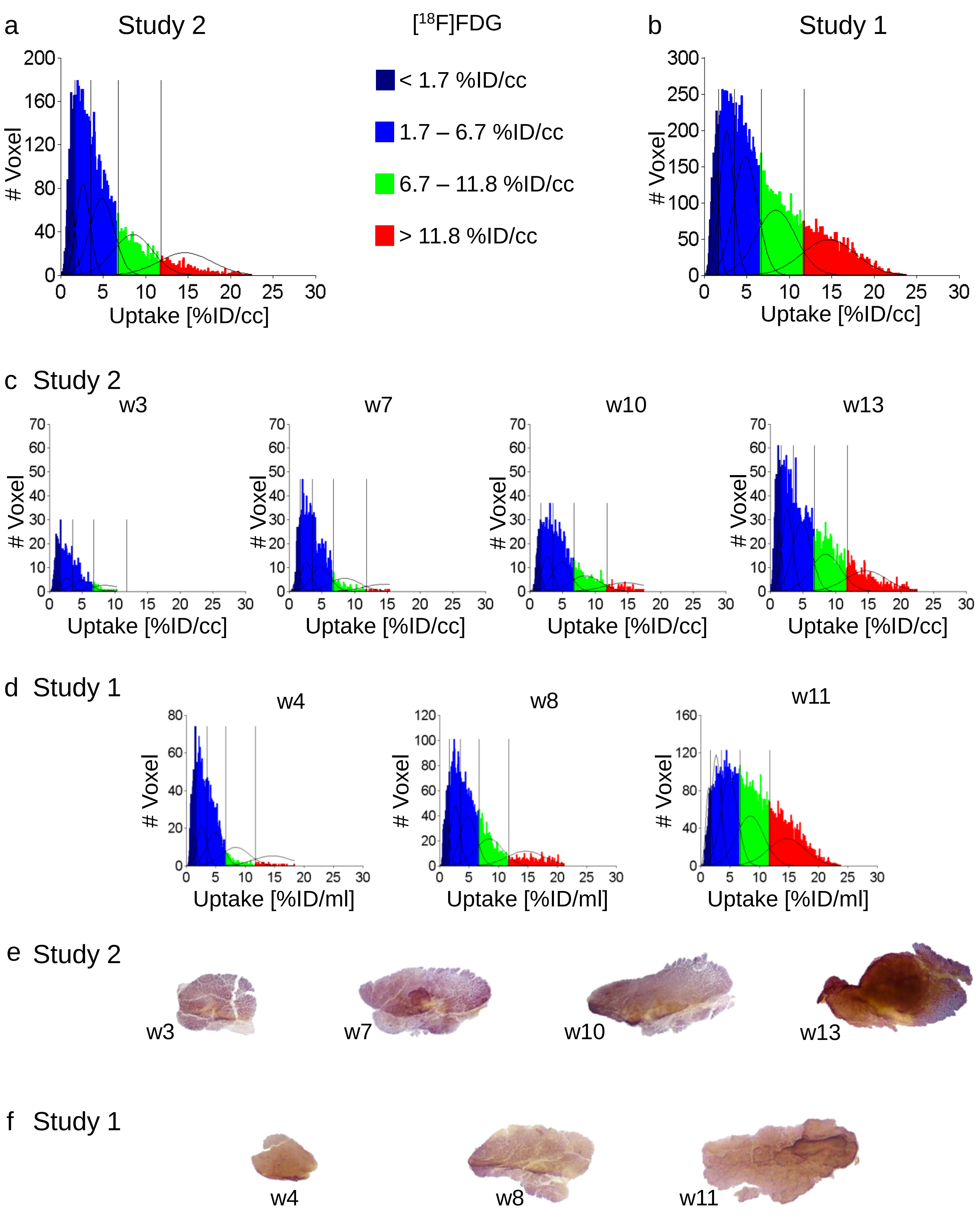

Supplemental Figure S3: Validation of the GMM analysis of [^{18}F]FDG in the MIN-O model: (a) The calculated GMM model of the training data set in study 1, including the thresholds, was applied to the summed histogram of study 2 as the test data set. (b) The GMM model of study 1 compared to the results in study 2 showed higher amounts of moderate (green; 6.7 – 11.8%ID/cc) and high uptake populations (red; > 11.8%ID/cc). (c, d) GMM results were applied to the single time points in study 2 (c) and study 1 (d), showing good agreement of both studies with a slightly slower appearance and lower amounts of the moderate and high uptake populations (green and red) in study 2. (e, f) Whole-mounts of representative lesions in study 2 (e) and study 1 (f) verified the slightly slower but comparable tumor development in study 2 compared to study 1.

Supplemental Fig. S4

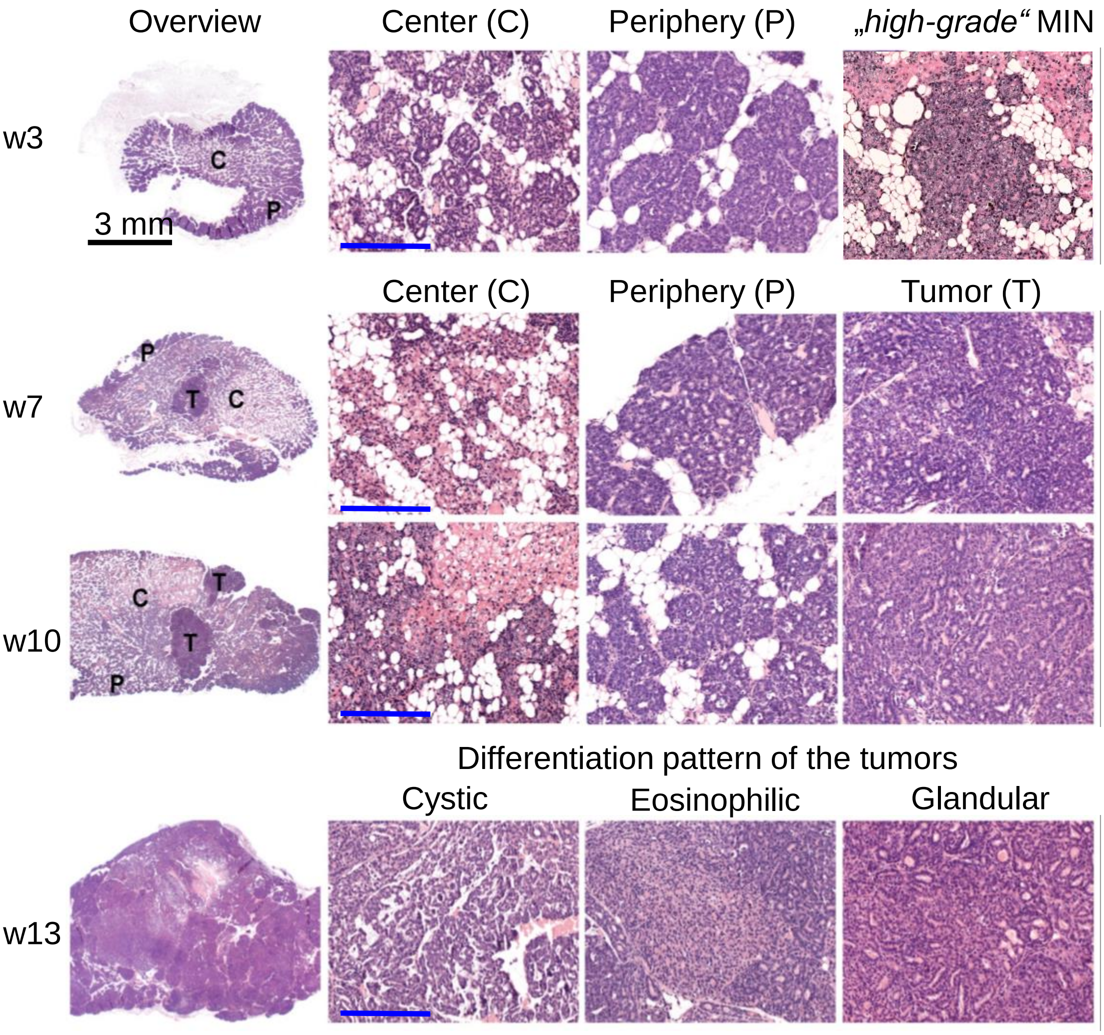

Supplemental Figure S4: Pathological description of tumor development in the MIN-O model: An overview of transplanted MIN-O tissues showing the development of lesions from hyperplastic outgrowth to IC from w3 until w13. In the center (C) of the hyperplastic lesions, low-grade MIN patterns with high differentiation were observed, whereas DCIS-like high-grade MIN regions with less differentiation were located in the periphery (P) of lesions from w3 until w10. In w7 (n = 1) and w10 (all lesions), additional solid tumor nests, arising from the center of the lesions, appeared that showed a glandular differentiation pattern. Only in w13 were large tumors observed, comprised of cystic, eosinophilic, and glandular differentiation patterns. The blue scale bar indicates 200 µm.

Supplemental Fig. S5

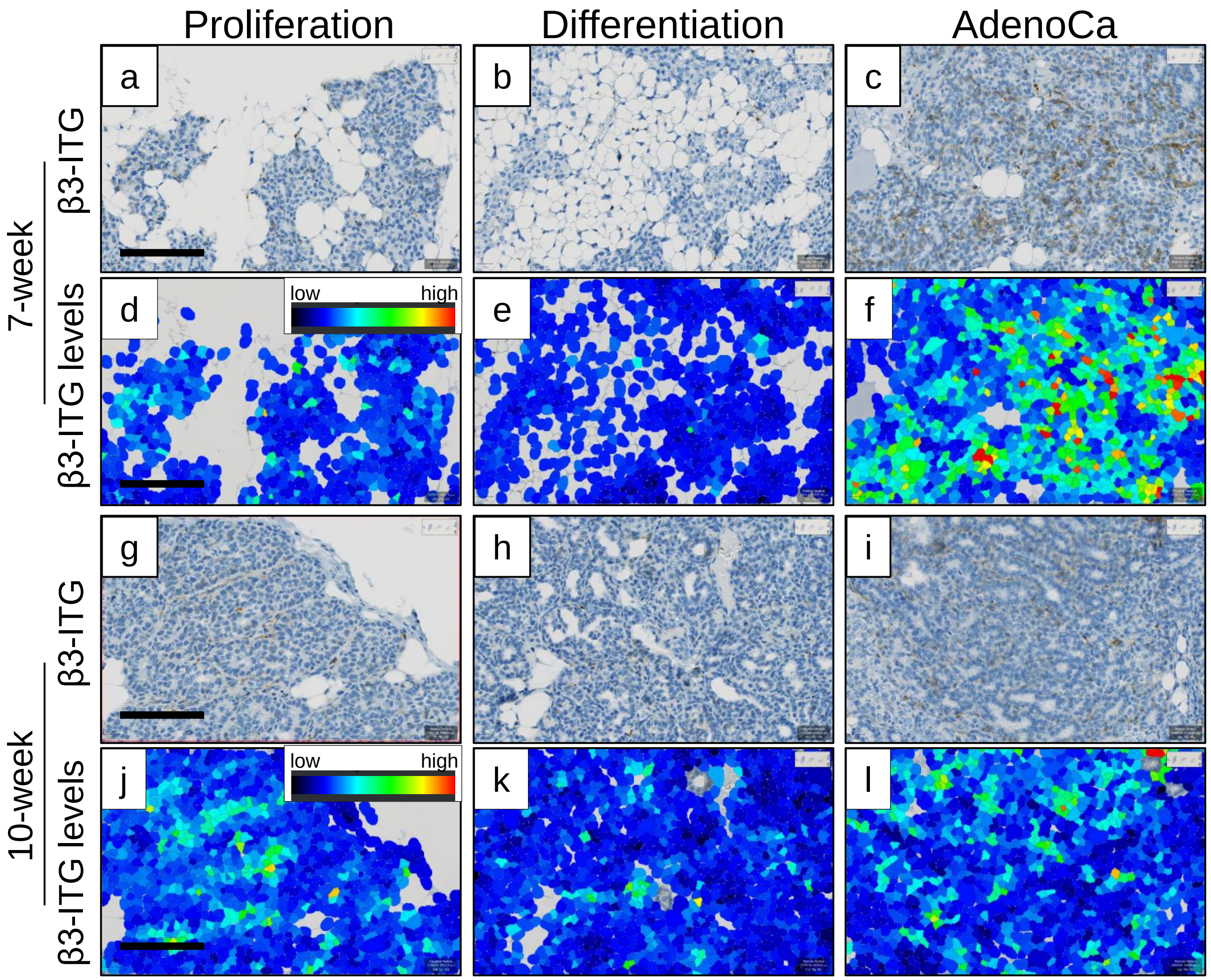

Supplemental Figure S5: β_3-Integrin expression: β_3-integrin stained MIN-O tissues at 7-week (a-f) and 10-week (g-l) and the levels of β_3-integrin (d-f, j-l) are shown. Images were analyzed at growth zone (a,d,g,j), differentiation zones (b,e,h,k) and tumor areas (indicated as AdenoCa; c,f,i,l). The color indicates the levels of β_3-integrin (d-f, j-l) as brighter color as higher β_3-integrin levels and dark color as lower β_3-integrin levels. The scale bar indicates 100 µm.

Supplemental Fig. S6

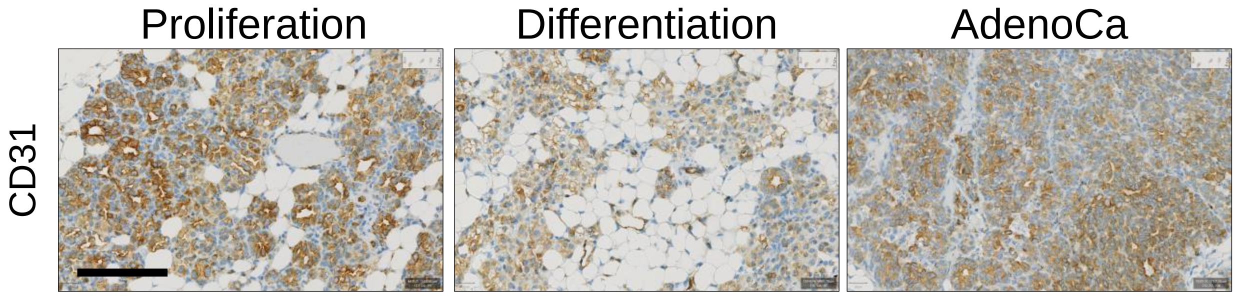

Supplemental Figure S6: Vessel density: Representative CD31 staining of 7-week MIN-O tissues showed a dense but heterogeneous blood vessel system within the proliferation and differentiation zones as well as in the adenocarcinomas. Positive CD31 staining in endothelial cells indicates possible vasculogenic mimicry in MIN-O tissues. The scale bar indicates 100 µm.

Supplemental Table 1: Uptake of [^{18}F]FDG in breast cancer patients.

	Tumor		DCIS	
**Patient**	**SUV max**	**SUV mean**	**SUV max**	**SUV mean**
1	8.77	7.39	1.27	1.22
2	16.05	12.95	1.37	1.31
